# Radiological Characterization of Cerebral Phenotype in Newborn Microcephaly Cases from 2015 Outbreak in Brazil

**DOI:** 10.1371/currents.outbreaks.e854dbf51b8075431a05b39042c00244

**Published:** 2016-06-08

**Authors:** Yuri Raoni Ramalho Rocha, José Ricardo Cavalcanti Costa, Pericles Almeida Costa, Gessica Maia, Rafael de Medeiros Vasconcelos, Cynthia Ramos Tejo, Rafaella Martins Batista, Manoel Lima Neto, Gustavo Graco Martins de Lima, Francisco Negromonte, Marcelle Borba, Selma Maria Bezerra Jeronimo, Eduardo Bouth Sequerra, Manuel Moreira Neto

**Affiliations:** Radiologia e Diagnóstico por Imagem, Hospital Universitário Onofre Lopes, Universidade Federal do Rio Grande do Norte, Natal, Rio Grande do Norte, Brazil; Radiologia e Diagnóstico por Imagem, Hospital Universitário Onofre Lopes, Universidade Federal do Rio Grande do Norte, Natal, Rio Grande do Norte, Brazil; Radiologia e Diagnóstico por Imagem, Hospital Universitário Onofre Lopes, Universidade Federal do Rio Grande do Norte, Natal, Rio Grande do Norte, Brazil; Radiologia e Diagnóstico por Imagem, Hospital Universitário Onofre Lopes, Universidade Federal do Rio Grande do Norte, Natal, Rio Grande do Norte, Brazil; Radiologia e Diagnóstico por Imagem, Hospital Universitário Onofre Lopes, Universidade Federal do Rio Grande do Norte, Natal, Rio Grande do Norte, Brazil; Radiologia e Diagnóstico por Imagem, Hospital Universitário Onofre Lopes, Universidade Federal do Rio Grande do Norte, Natal, Rio Grande do Norte, Brazil; Radiologia e Diagnóstico por Imagem, Hospital Universitário Onofre Lopes, Universidade Federal do Rio Grande do Norte, Natal, Rio Grande do Norte, Brazil; Radiologia e Diagnóstico por Imagem, Hospital Universitário Onofre Lopes, Universidade Federal do Rio Grande do Norte, Natal, Rio Grande do Norte, Brazil; Radiologia e Diagnóstico por Imagem, Hospital Universitário Onofre Lopes, Universidade Federal do Rio Grande do Norte, Natal, Rio Grande do Norte, Brazil; Radiologia e Diagnóstico por Imagem, Hospital Universitário Onofre Lopes, Universidade Federal do Rio Grande do Norte, Natal, Rio Grande do Norte, Brazil; Radiologia e Diagnóstico por Imagem, Hospital Universitário Onofre Lopes, Universidade Federal do Rio Grande do Norte, Natal, Rio Grande do Norte, Brazil; Institute of Tropical Medicine of Rio Grande do Norte, Federal University of Rio Grande do Norte, Natal, RN, Brazil; Department of Biochemistry, Federal University of Rio Grande do Norte, Natal, RN, Brazil; Institute of Science and Technology of Tropical Diseases, Brazil; Brain Institute, Universidade Federal do Rio Grande do Norte, Natal, Rio Grande do Norte, Brazil; Radiologia e Diagnóstico por Imagem, Hospital Universitário Onofre Lopes, Universidade Federal do Rio Grande do Norte, Natal, Rio Grande do Norte, Brazil

**Keywords:** Brazil, computed tomography, microcephaly, Outbreak, zika virus

## Abstract

Introduction: Brazil is facing, since October of 2015, an outbreak of microcephalic fetuses. This outbreak is correlated with the beginning of circulation of Zika virus (ZIKV) in the country. Although it is clear that the size of the head is diminished in these fetuses, the brain phenotype associated with these malformations is unknown.

Methods: We collected computed tomography images of the microcephaly cases from the region of Natal, Rio Grande do Norte, from September 2015 to February 2016.

Findings: The microcephalies derived from the current outbreak are associated with intracerebral calcifications, malformation of the ventricular system, migratory disorders in the telencephalon and, in a lower frequency, malformation of the cerebellum and brainstem.

Discussion: The characteristics described herein are not usually found in other types of microcephaly. We suggest that this work can be used as a guideline to identify microcephaly cases associated to the current outbreak.

## INTRODUCTION

Microcephaly is a congenital malformation caused by a reduced brain growth, which leads to diminished growth of the head circumference. The diagnosis is defined by a cephalic perimeter of 2 standard deviations (SD) below average for age and gender.^1^ Microcephaly can have a genetic cause or be a consequence of environmental factors, especially congenital infections, such as virus, bacteria, protozoan or fungi.^2^

Since October of 2015, an outbreak of microcephaly started to be reported in Brazil,^3^ coinciding with previous introduction of ZIKV in the country, estimated to have happened sometime in 2014^4^^,^ 5 and with its outbreak in the beginning of 2015.^6^ ZIKV was first detected in amniotic fluid of microcephalic fetuses from Campina Grande, Paraíba. Presence of ZIKV was also detected in blood and tissue samples from a newborn from Ceará that died right after birth and had multiple malformations, including microcephaly. ZIKV was also detected in placental analysis of a fetus aborted in the city of Natal, Rio Grande do Norte.^7^ These combined findings suggest that the virus can cross the placental barrier in humans. Later, a group in Slovenia detected ZIKV in a fetus of a pregnant woman that was infected in Natal, returned to her home country and interrupted the pregnancy at 32 weeks of gestation, the fetus was microcephalic.^8^ Together, these data, although preliminary, suggest a strong correlation between ZIKV congenital infection and the development of microcephaly.

ZIKV is a flavivirus that was first described in Uganda.^9^ On one of its earliest descriptions, ZIKV was described to be able to infect neural cells when intracerebrally injected in mice.^10^ In these mice, ZIKV remained on the neural tissue and was only recovered from it, suggesting a neural tropism.^10^ In humans though, the symptoms described did not indicate any neural tropism until a correlation with Guillain-Barré syndrome was first reported during an outbreak in French Polynesia on 2013.^11^^,^ 12 Phylogentic analysis reveals that ZIKV circulating in Brazil comes from French Polynesia.^13^ Therefore, it is possible that the strain from French Polynesia became more neurotropic.

According to Brazil’s Health Ministry, until January 1st, 2016, there were 3174 new cases of microcephaly, in 684 cities of 21 states across the country. The greater incidence was registered in Pernambuco, in the Brazilian northeast, with a total of 1185 new cases. The state of Rio Grande do Norte, whose records between 2010 and 2014 had shown an annual average of 1,8 cases/year, showed a total of 169 new cases in 2015, with 11 deaths.^14^ In this study, we describe the cranial phenotype of 27 children with microcephaly from the state of Rio Grande do Norte, who were examined by computed tomography.

## METHODS

A retrospective, observational, transversal study was performed to evaluate the cranial computed tomography (CT) findings from patients (n=27) with microcephaly. These data comes from every single child with clinical diagnosis of microcephaly studied at Hospital Universitário Onofre Lopes (HUOL). HUOL is the reference hospital in the state of Rio Grande do Norte for microcephaly cases. The cases used here occurred between September 2015 and February 2016.

The CT acquisition was performed by a multidetector equipment, Philips Brilliance 64 (Philips Medical Systems; Cleveland, EUA), with tomographic section thickness of 1.0 mm, without administration of iodinated contrast.

The protocol for this study was reviewed and approved by the Universidade Federal do Rio Grande do Norte Ethics Committee, certificate of approval CAAE 53111416.7.0000.5537.

The cases of microcephaly were evaluated by two radiologists with experience in pediatric neuroradiology, who interpreted the following findings: 1. Intracranial calcifications, distinguishing their distribution between subcortical, in the basal ganglia and/or periventricular regions; 2. Ventricular system dilation, defined as an qualitative analysis alteration leading to enlargement of the dimensions of the lateral ventricles in relation to the cranial biparietal diameter; 3. Ventricular morphological alteration, considering cases with dismorphism or significative assimetry of lateral ventricles; 4. Neuronal migration disorder, differentiating patterns of lissencephaly - most of the brain presenting smooth surface without sulci, or paquigyria - a pattern consisting of wide gyri with shallow and scarce sulci; 5. Malalignment of cranial bones, evidenced by a considerable degree of angulation or overlap between bones, promoting loss of the convex aspect of the skullcap; 6. Cerebellar hypoplasia/atrophy; 7. Brainstem atrophy, evidenced by a qualitative analysis of the volumetrical decrease of these structures with prominence of liquor spaces in the posterior fossa; 8. Cerebral white matter cysts, identified as structures with liquor density in the brain parenchyma; 9. Ocular globe alteration, demonstrated by the presence of calcifications or anormality of its habitual anatomy.

## RESULTS

**Figure 1:**
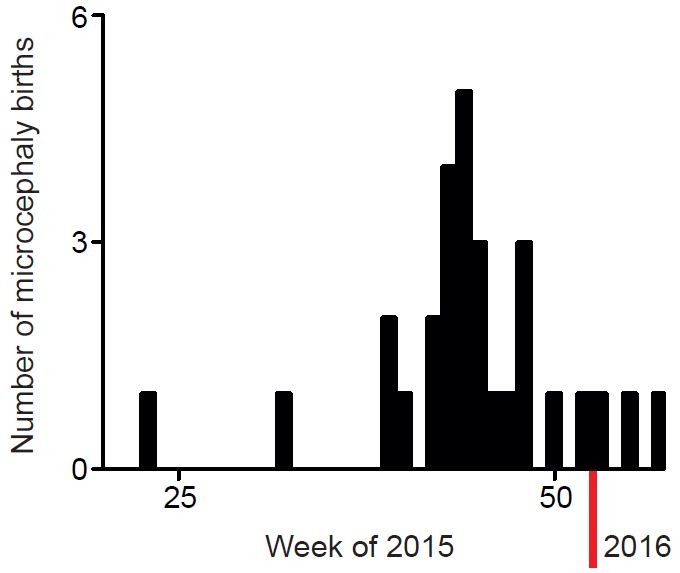
**Figure 1.** Dates of birth of microcephaly cases registered at Hospital Onofre Lopes after May 2015.

This group of patients is composed of 14 females and 13 males. Patient’s age during imaging varied from 5 days to 4 months of age. The dates of birth of microcephalic patients from May 2015 to February 2016 are shown in Figure 1. The patients selected for analysis here are those that were born after September 2015, when the frequency of cases started to increase (Figure 1).

**Figure 2:**
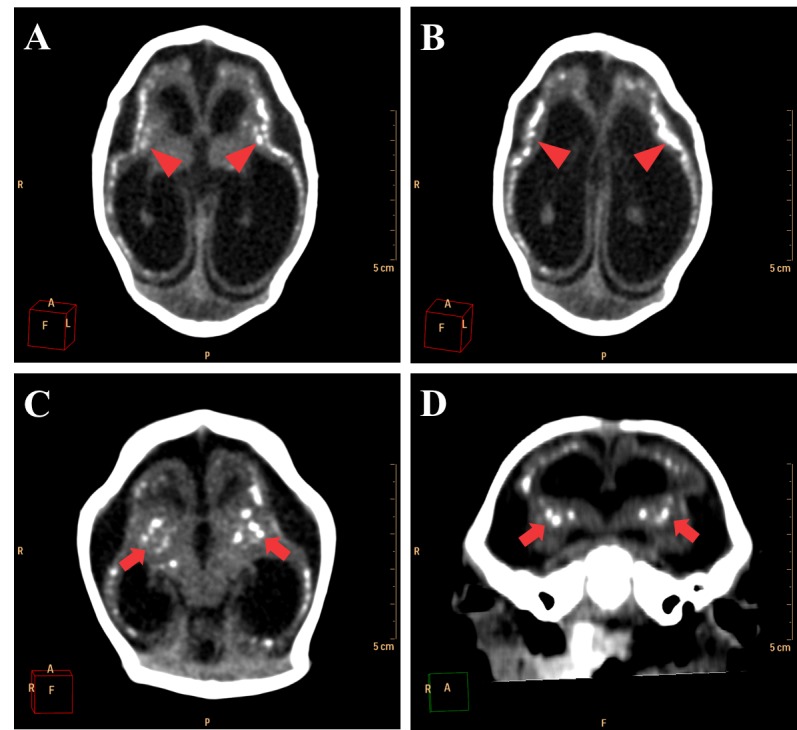
**Figure 2.** Most frequent radiological findings on CT. Axial (A, B and C) and coronal (D) non-contrast brain CT. A and B show a 1 month old infant female with evidence of severe microcephaly with subcortical gross calcifications (red arrowheads), accentuated compensatory lateral ventricles dilation and loss of typical sulcation configuring lisencephaly. (C and D) Gross calcifications in the basal ganglia (red arrows) in a 2 months old female newborn, configuring one of the most typical findings of this presentation.

The major radiological findings are shown in Table 1. Intracranial calcifications, ventricular system dilation and neuronal migration disorders were present in all 27 cases. In contrast, ocular globe alterations were not found in any of the radiologic studies. Intracranial calcifications (Figure 2) were found predominantly in subcortical regions, which were observed in 25 cases (92,6%), in basal ganglia in 20 cases (74,1%) and periventricular in 18 cases (66,7%).

**Table 1. Radiological findings:** 

Finding	Number of patients	Percentage
Intracranial calcifications	27	100.0%
Ventricular system dilation	27	100.0%
Neuronal migration disorder	27	100.0%
Ventricular morphological alteration	24	88.9%
Cranial bones malalignment	17	63.0%
Cerebellar atrophy/hypoplasia	12	44.4%
Brainstem atrophy	10	37.0%
Cerebral White matter cysts	8	29.6%
Ocular globe alteration	0	0

**Figure 3:**
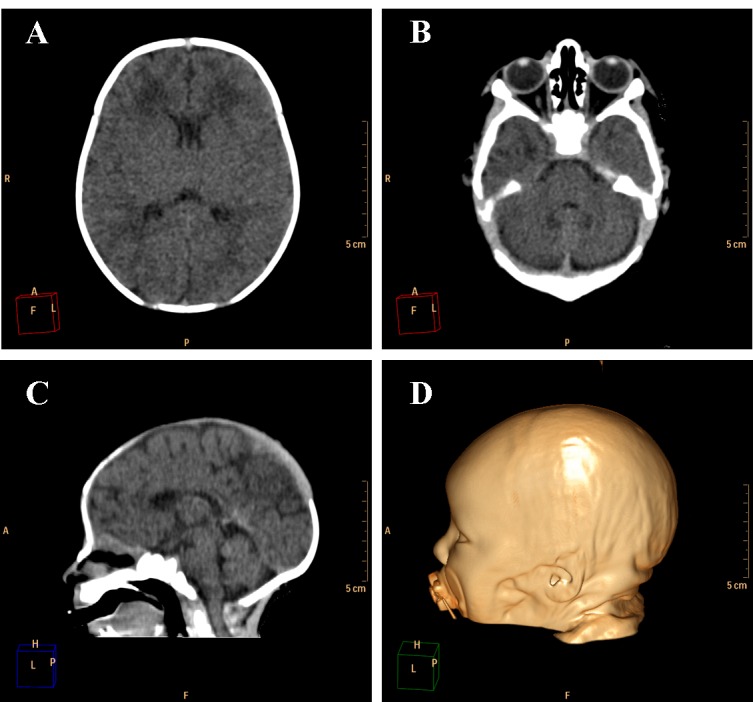
**Figure 3. **Example of a normally developed 10 days old newborn. A and B. Axial images of the brain showing the lateral ventricles and the cerebellum with its regular dimensions. C. Sagittal image showing regular size of the brain structures and alignment of the skull bones. D. Volume rendering of the head of the patient showing regular cranio-facial proportion.

**Figure 4:**
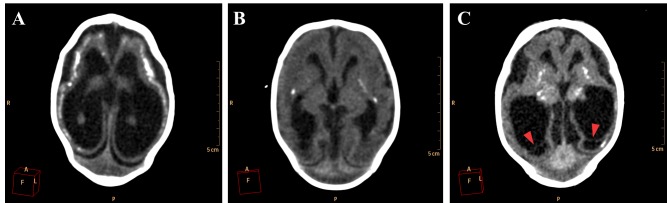
**Figure 4.** Axial non-contrast brain CT with three different patterns of parenchymal atrophy and compensatory ventricular dilation. Ventricular adherences are suggested by the presence of thin septations with soft tissue density observed in the occipital horns of lateral ventricle (red arrowheads in C).

Ventricular dilation was observed in all cases, with a compensatory, non-hypertensive pattern, secondary to atrophy and volumetric decrease of cerebral parenchyma. These cases showed parenchymal atrophy varying from moderate to severe degrees, which we assume was the main determining factor for the development of microcephaly (Figure 4, see also Figure 3 for comparison). Besides alterations in the dimension of the ventricles, 24 cases also had alterations in the morphology of the ventricular system (88,9%).

**Figure 5:**
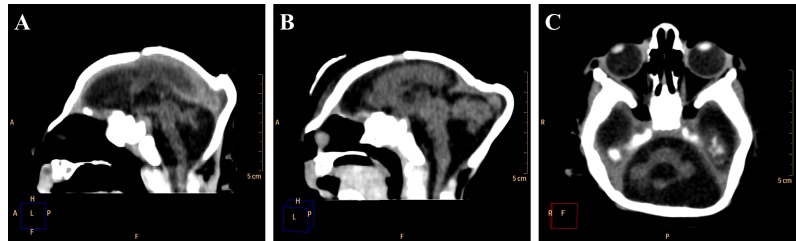
**Figure 5.** Sagittal (A and B) and axial (C) non-contrast brain CT showing cerebellar atrophy/hypoplasia.

Cerebellar atrophy/hypoplasia was observed in 12 cases (44,4%), while brainstem atrophy was detected in 10 patients (37,0%), with a more evident malformation of the pons. We also noted an association between these findings, since all patients who presented brainstem atrophy showed also cerebellar atrophy (Figure 5, see Figure 3B for a normal brainstem and cerebellum).

**Figure 6:**
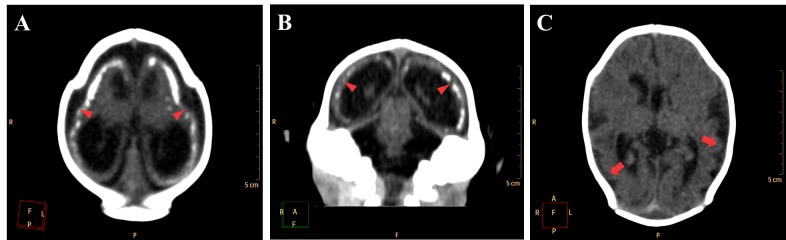
**Figure 6.** Neuronal migration disorders in female newborns in axial (A and C) and coronal (B) images, showing a pattern of lissencephaly/agyria (red arrowheads) and pachygyria (red arrows).

Neuronal migration disorders were observed in all 27 cases, especially the patterns of lissencephaly/agyria (smooth cortical surface), in 16 cases (59,3%), and pachygyria (broad gyri with scarce and plane sulci), in 11 cases (40,7%; Figure 6).

**Figure 7:**
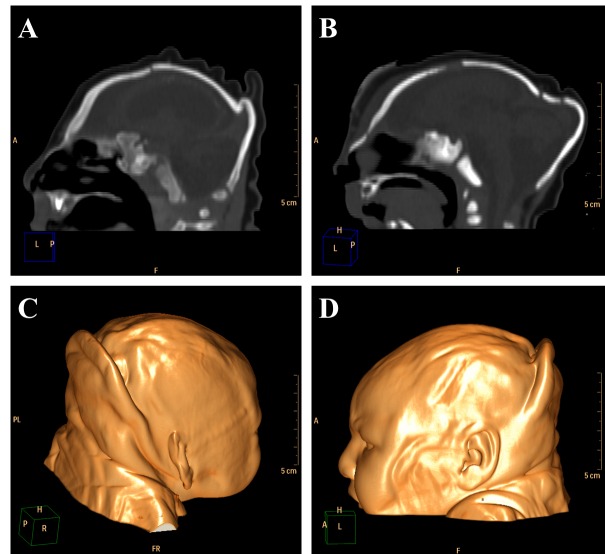
**Figure 7.** Sagittal sections present evidence of cranial bone overlap (A), frontal and occipital bones horizontalization (B), which could be observed clinically in association to cutaneous folds accentuation (Volume Rendering; C e D).

**Figure 8:**
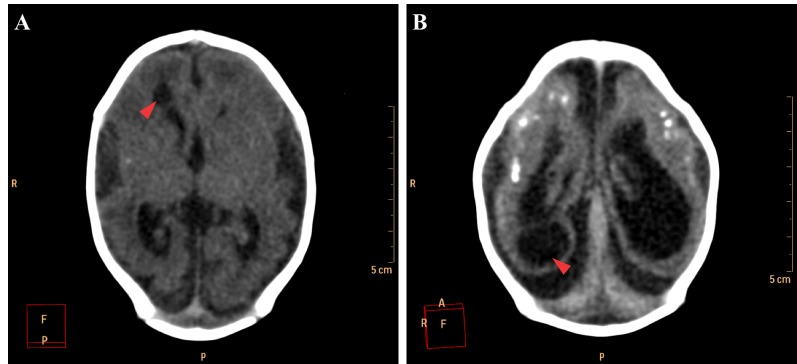
**Figure 8.** Cystic encephalomalacia in the periventricular white matter (red arrowheads) of the right lateral ventricle frontal horn (A) and adjacent to its occipital horn (B).

The encephalic mass reduction induced osseous alterations (Figure 7, see Figure 3C and D for the normal phenotype), as seen in several patients with bone malalignment, particularly parietal over the occipital bones, present in 17 cases (63,0%). Eight (29,6%) patients displayed white substance cystic lesions, an alteration compatible with leukomalacia secondary to the infectious insult, and, when present, it had a periventricular pattern (Figure 8). Corpus callosum dysgenesia was an additional finding in five patients, but did not correlate with the degree of parenchymal loss.

**Figure 9:**
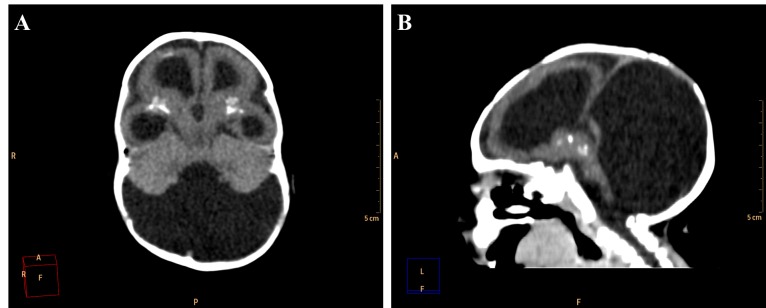
**Figure 9.** Axial (A) and sagittal (B) non-contrast brain CT show a 3 month old infant female evidencing hypoplastic cerebellar vermis, cystic dilatation of the fourth ventricle and enlargement of the posterior fossa, representing Dandy-Walker syndrome.

Among the cases evaluated, we found one with malformation of Dandy-Walker in association, whose findings are hypoplastic cerebellar vermis, cystic dilatation of the fourth ventricle and enlargement of the posterior fossa^15^ (Figure 9). Along with these characteristics, the patient also has periventricular and basal ganglia calcifications and lissencephaly.

## DISCUSSION

The clinical entities classically related to congenital microcephaly of acquired aetiology are infections that compose the TORCH complex, a group of agents that includes Toxoplasmosis, "Others" (syphilis, Varicella-Zoster virus, Parvovirus B19, Hepatitis B), Rubella, Cytomegalovirus and Herpes simplex virus.^16^ The sudden growth of microcephaly cases in Brazil, apparently not attributed to these classical agents, has a strong correlation with an outbreak of ZIKV. The cranioencephalic malformations in TORCH-congenitally-infected fetuses are very similar, and subtle specific characteristics are used to a better etiologic definition. In the present study, we evaluated by CT the heads of 27 patients with microcephaly, presumably due to ZIKV infection; we found a tomographic pattern distinct from microcephalies induced by classically described defects. Toxoplasmosis and CMV infections cause the most similar phenotypes to the ones described here. In toxoplasmosis-induced microcephalies, diffuse intracranial calcifications are usually associated with calcification of the ocular globes and/or hydrocephally, usually caused by reduction of cerebral spine fluid flow secondary to aqueduct stenosis. In the present cases, hydrocephaly is caused by a compensation of the loss of parenchymal volume. While cases due to toxoplasmosis infections have diffuse calcifications, our study brought cases which showed predominantly subcortical calcifications. Besides, we could not find ocular globe alterations in these patients. The most similar phenotype of microcephalies is induced by CMV, in which the intracranial calcifications are located close to the ventricular surface and are associated with migration disorders. Although we have seen migration disorders very similar to the ones found in CMV-derived cases, the calcification pattern is clearly different.

The population described herein has some distinctions from a microcephalic population described previously. Schuler-Faccini and collaborators^17^ reported a group of 27 newborns with microcephaly, presumably associated to ZIKV, describing some CT and transfontanellar ultrasound findings. They detailed a pattern of generalized intracranial calcifications, specially periventricular, on brain parenchyma, thalamus and basal ganglia, with a prevalence of 74% (20 cases); 33% (9 patients) of newborns showed neuronal migration anomalies (lissencephaly, pachygyria); and ventricular enlargement secondary to encephalic atrophy was described in 44% (12 cases).^17^ The prevalence of these findings was lower than those found in the present population from Rio Grande do Norte. The population shown here shows 100% (27) of the patients with calcifications, migration disorders and ventricular enlargement. In Schuler-Faccini et al. report^17^, the geographic location of the patients was not included in the article, making it difficult to determine whether there is any regional effect. However, as the imaged patients represent the most severe phenotypes in their study (with 3SD below the mean head circumference) they were expected to find an incidence of disorders similar or worse than the one presented here. As the number of cases of microcephaly in our state was very low previously to the current outbreak (1.8 per year 14), we expected to have a homogeneous population in terms of cause of malformations.

The several alterations observed in such microcephaly cases still require a confirmation for a supposed cause-effect relation with the ZIKV infection, and it will be necessary to elucidate the sequence of biological events that impact development of the CNS. Two recent papers report that the African Zika isolate from 1947 is capable of infecting human neural stem cells in vitro.^18^^,^ 19

This evidence of the pathogenics generating a reduction of the population of neuronal progenitor cells can explain the degree of encephalic atrophy seen in microcephaly cases and suggests the possibility of greater severity in early infected fetuses during in utero development, once there is a major proportion of progenitor cells in such phase of prenatal growth.

## CONCLUSION

The phenotype of the microcephaly cases associated with the current outbreak at Rio Grande do Norte state is more severe and different from previous cases. The data here presented can be used as a guideline for those who want to differentiate microcephalies caused by the probable ZIKV-associated outbreak. The consequences of this type of microcephalies to the children, their families and the public health system are still to be determined.

## COMPETING INTERESTS

The authors declare having no competing interests.

## DATA AVAILABILITY STATEMENT

The data presented in this article is freely available from FigShare.

## References

[1] Ashwal S, Michelson D, Plawner L, Dobyns WB. Practice parameter: evaluation of the child with microcephaly (an evidence-based review). Neurology 2009; 73:887–97. 10.1212/WNL.0b013e3181b783f7 PMC2744281 19752457

[2] Epps RE, Pittelkow MR, Su WP. TORCH syndrome. Sem Dermatol 1995; 14:179-86. 10.1016/s1085-5629(05)80016-1 7640200

[3] Brasil. Ministério da Saúde (2015, Nov 28). Ministério da Saúde confirma relação entre vírus zika e casos de microcefalia. http://www.brasil.gov.br/saude/2015/11/ministerio-da-saude-confirma-relacao-entre-virus-zika-e-microcefalia

[4] Musso D. Zika virus transmission from French Polynesia to Brazil. Emerg Infect Dis 2015; 21:1887. 10.3201/eid2110.151125 PMC4593458 26403318

[5] Zammarchi L, Tappe D, Fortuna C, et al. Zika virus infection in a traveller returning to Europe from Brazil, March 2015. Euro Surveill 2015; 20:pii=21153. 10.2807/1560-7917.es2015.20.23.21153 26084316

[6] Kindhauser MK, Allen T, Frank V, Santhana R, Dye C. Zika: the origin and spread of a mosquito-borne virus. Bull World Health Org 2016; published online. 10.2471/BLT.16.171082 PMC5034643 27708473

[7] Martines RB, Bhatnagar J, Keating K, et al. Notes from the field: Evidence of Zika virus infection in brain and placental tissues from two congenitally infected newborns and two fetal losses- Brazil, 2015. MMWR Morb Mortal Wkly Rep 2016; 65:150-60. 10.15585/mmwr.mm6506e1 26890059

[8] Mlakar J, Korva M, Tul N, et al. Zika vírus associated with microcephaly. N Engl J Med 2016; 374:951-8. 10.1056/NEJMoa1600651 26862926

[9] Dick GWA, Kitchen SF, Haddow AJ. Zika virus (I). Isolations and serological specificity. Trans Roy Soc Trop Med and Hyg 1952; 46:509-520. 10.1016/0035-9203(52)90042-4 12995440

[10] Dick GWA. Zika virus (II). Pathogenicity and physical properties. Trans Roy Soc Trop Med and Hyg 1952; 46:521-534. 10.1016/0035-9203(52)90043-6 12995441

[11] Oehler E, Watrin L, Larre P, Leparc-Goffart I, Lastere S, Valour F, Baudoin L, Mallet HP, Musso D, Ghawche F. Zika virus infection complicated by Guillain-Barré syndrome - case report, French Polynesia, December 2013. Euro Surveill 2014; 19: pii=20720. 10.2807/1560-7917.es2014.19.9.20720 24626205

[12] Cao-Lorneau V, Blake A, Mans S, et al. Guillain-Barré syndrome outbreak associated with Zika virus infection in French Polynesia: a case-control study. Lancet, in press. 10.1016/S0140-6736(16)00562-6 PMC5444521 26948433

[13] Calvet G, Aguiar RS, Melo ASO, et al. Detection and sequencing of Zika virus from amniotic fluid of fetuses with microcephaly in Brazil: a case study. Lancet Infect Dis, in press. 10.1016/S1473-3099(16)00095-5 26897108

[14] Brasil. Ministério da Saúde (2016, Jan 1). Ministério da Saúde atualiza casos suspeitos de microcefalia. http://portalsaude.saude.gov.br/index.php/cidadao/principal/agencia-saude/21459-saude-divulga-dados-atualizados-de-microcefalia,

[15] Ecker JL, Shipp TD, Bromley B, Benacerraf B. The sonographic diagnosis of Dandy–Walker and Dandy–Walker variant: Associated findings and outcomes. Prenat Diagn. 2000; 20:328–32. 10740206

[16] Yadav RK, Maity S, Saha S. A review on TORCH: groups of congenital infection during pregnancy. J Sci In Res 2014; 3:258-264.

[17] Schuler-Faccini L, Ribeiro EM, Feitosa IM, et al. Possible association between zika virus infection and microcephaly – Brazil, 2015. MMWR Morb Mortal Wkly Rep 2016; 65:59-62. 10.15585/mmwr.mm6503e2 26820244

[18] Tang H, Hammack C, Ogden SC, et al. Zika virus infects human cortical neural progenitors and attenuates their growth. Cell Stem Cell 2016; 18:1-4. 10.1016/j.stem.2016.02.016 PMC5299540 26952870

[19] Garcez PP, Loiola EC, Madeiro da Costa RF, et al. Zika virus impairs growth in human neurospheres and brain organids. Science 2016; 352 (6287): 816-818. 10.1126/science.aaf6116 27064148

